# The accuracy of 3D virtual bone models of the pelvis for morphological sex estimation

**DOI:** 10.1007/s00414-019-02002-7

**Published:** 2019-01-24

**Authors:** Kerri L. Colman, Alie E. van der Merwe, Kyra E. Stull, Johannes G. G. Dobbe, Geert J. Streekstra, Rick R. van Rijn, Roelof-Jan Oostra, Hans H. de Boer

**Affiliations:** 1 Department of Medical Biology, Section Clinical Anatomy and Embryology, Amsterdam UMC, location AMC, University of Amsterdam, Meibergdreef 9, Amsterdam, The Netherlands; 2 Department of Anthropology, University of Nevada, Reno, Reno, Nevada USA; 3 Faculty of Health Sciences, Department of Anatomy, University of Pretoria, Pretoria, South Africa; 4 Department of Biomedical Engineering and Physics, Amsterdam UMC, location AMC, University of Amsterdam, Meibergdreef 9, Amsterdam, The Netherlands; 5 Department of Radiology, Amsterdam UMC, location AMC, University of Amsterdam, Meibergdreef 9, Amsterdam, The Netherlands; 6 Department of Pathology, Amsterdam UMC, location AMC, University of Amsterdam, Meibergdreef 9, Amsterdam, The Netherlands; 7 Department of Forensic Medicine, Netherlands Forensic Institute, The Hague, The Netherlands

**Keywords:** Radiology, Virtual anthropology, Accuracy, Reliability, Sex estimation, Pelvis

## Abstract

It is currently unknown whether morphological sex estimation traits are accurately portrayed on virtual bone models, and this hampers the use of virtual bone models as an alternative source of contemporary skeletal reference data. This study determines whether commonly used morphological sex estimation traits can be accurately scored on virtual 3D pelvic bone elements. Twenty-seven intact cadavers from the body donation program of the Amsterdam UMC, University of Amsterdam, were CT scanned; this data was used to produce virtual bone models. Thereafter, the dry bones were obtained. Three traits by Klales (2012) and five traits from the Workshop of European Anthropologists (WEA) (1980) were scored on the virtual bone models and their dry skeletal counterparts. Intra- and inter-observer agreement and the agreement between the scores for each virtual bone model-dry bone pair were calculated using weighted Cohen’s kappa (K). For all Klales (2012) traits, intra- and inter-observer agreement was substantial to almost perfect for the virtual- and dry bones (K = 0.62–0.90). The agreement in scores in the virtual-dry bone pairs ranged from moderate to almost perfect (K = 0.58–0.82). For the WEA (1980) traits, intra-observer agreement was substantial to almost perfect (K = 0.64–0.91), but results were less unambiguous for inter-observer agreement (K = 0.24–0.88). Comparison of the scores between the virtual bone models and the dry bones yielded kappa values of 0.42–0.87. On one hand, clinical CT data is a promising source for contemporary forensic anthropological reference data, but the interchangeability of forensic anthropological methods between virtual bone models and dry skeletal elements needs to be tested further.

## Introduction

Sex estimation is a key component for creating a forensic anthropological biological profile as the estimation of the other biological profile elements (age at death, stature, and ancestry) relies heavily on the accurate estimation of sex. Mainly due to differences in reproductive function the pelvis is considered to be the most sexually dimorphic skeletal element in humans and therefore the most reliable skeletal element for sex estimation [1–7]. Both metric and morphological sex estimation methods exist, but the latter is often favored because of the ease of application and applicability across populations and time [8–10].

In order to be considered acceptable as evidence in a Court of Law, the accuracy of forensic anthropological sex estimation methods need to be known [11, 12]. For this, they should be derived from or tested on large and representative skeletal populations. Large known skeletal populations exist in numerous parts of the world (e.g. the USA, South Africa, Thailand) and have been used in development and validation studies [7, 13, 14]. However, in most European countries, no contemporary collections exist [15–20]. Specifically in the Netherlands, due to legislation and the high mean age of bodies donated to the body donation programs in medical schools [21, 22], it is impossible to compile a skeletal collection that is representative of the current population in the Netherlands.

Radiological data, such as computed tomography (CT) scans, derived from a clinical/hospital setting, might be used as a proxy since the number of scans generated on a daily basis guarantees a large, contemporary and representative source of data. Data acquired from a clinical radiological setting is actually a more accurate representation of the current population than most body donation programs since it is less affected by secular trends and age bias. Furthermore, reconstructions that are based on the CT scan data can provide a precise three-dimensional (3D) model that represents the original skeletal element [23]. However, in order for CT scans to be a feasible alternative to traditional skeletal collections, the morphological traits indicating sex also need to be accurately portrayed in the 3D reconstructions and their associated sex estimation scores need to correspond to the scores recorded on the dry skeletal element.

Some studies have already explored whether morphological methods can be applied on radiological data sources, however, the traits investigated were primarily scored on a binary scale (i.e. absent/present scores) [24, 25]. It therefore remains uncertain if the often-subtle morphological features defined on ordinal scales (scales of 1 to 5 or − 2 to 2) can be scored accurately on virtual bone models. Consequently, one cannot readily assume that morphological sex estimation methods that are traditionally applied to dry skeletal remains perform equally when applied to virtual 3D skeletal elements.

The current study aims to determine whether commonly used morphological sex estimation methods that utilize ordinal scales can be accurately scored on reconstructed virtual 3D pelvic bone elements.

## Material and methods

**Fig. 1:**
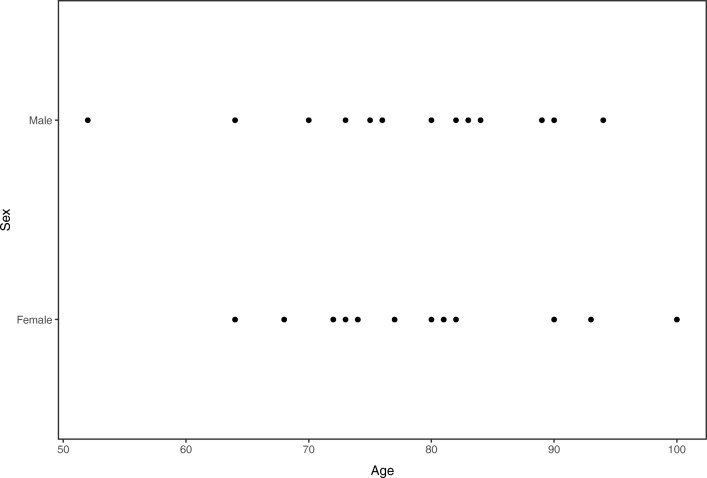
Demographic profile of the 27 (13 males, 14 females) fully intact cadavers included in this study

Twenty-seven (13 males, 14 females) fully intact cadavers were randomly selected from the body donation program of the Amsterdam Universitair Medische Centra (UMC), University of Amsterdam, Department of Medical Biology, section Clinical Anatomy and Embryology. The mean age at death was 78 years (range 52–94 years) for males and 80 years (range 64–100 years) for females (Fig. 1).

All cadavers were CT scanned while fully fleshed using a standard patient scanning protocol (120 kV, 150 mAs, slice thickness 0.9 mm, increment 0.45 mm, reconstruction kernel D (bone filter)) on a Philips Brilliance 64 (Philips Medical Systems, Best, The Netherlands). The scan data were used to segment 3D virtual bone models of the os coxae using dedicated in-house research software. Specifics regarding this software package and segmentation process can be found in Dobbe (2011) [26] and Colman (2017) [23]. Following CT scanning, the bodies were macerated to obtain the dry os coxae by using a straightforward, yet effective, method of removing the majority of soft tissue and then submerging the os coxae into boiling water.

Morphological sex estimation traits, namely the ventral arc (VA), subpubic concavity (SPC) and the medial aspect of the ischial pubic ramus (MA), being the three Phenice traits expanded to an ordinal scale as described by Klales (2012) [8], and the pre-auricular sulcus (PAS), greater sciatic notch (GSN), pubic angle (PA), arc compose (AC), and the ischial body (IB) as described by the Workshop of European Anthropologists (WEA) (1980) [27, 28] were selected for investigation. These five WEA (1980) traits were selected due to the range of complexity that they provide. In addition, the selected traits are amongst the ones most commonly used for sex estimation in the anthropological community [13, 29–31]. All selected traits, both Klales (2012) and the specific WEA (1980) traits, were scored on both the virtual bone models and the dry bones. Scoring was performed using a five-tier hyper-feminine to hyper-masculine scale ranging between − 2 and 2 for the WEA (1980) characteristics and 1 to 5 for the features as described by Klales (2012). See WEA (1980) and or Klales (2012) for detailed descriptions associated with each score. The virtual bone models were scored using the aforementioned dedicated in-house research software, as well as MeshLab [32], an open source online commercial viewing software, to determine whether differences in software packages would result in different scores or accuracies. Scoring took place twice by two independent observers with a minimum of 1-week lapse between repeat observations.

Intra- and inter-observer agreement for scores on the virtual bone models and the dry bone counterparts (i.e., reliability), as well as the agreement between the scores for each virtual bone model-dry bone pair (i.e. accuracy), were calculated using Cohen’s (1968) weighted kappa (K) [33]. Levels of agreement were interpreted according to the thresholds defined by Landis and Koch (1977) [34]: K < 0 indicates less than chance agreement, K = 0.01 to 0.20 indicates slight agreement, K = 0.21 to 0.40 indicates fair agreement, K = 0.41 to 0.60 indicates moderate agreement, K = 0.61 to 0.80 indicates substantial agreement and K = 0.81 to 1.0 indicates almost perfect to perfect agreement. All statistical analyses were done using R, version 3.3.0.

## Results

### Sex estimation features based on the Klales (2012) traits

#### Intra- and inter-observer agreement

Cohen’s weighted kappa (K) values indicate substantial to almost perfect agreement for the intra- and inter-observer agreement for the three Klales (2012) traits when scored in isolation on the dry bones (K = 0.78–0.89 and 0.61–0.77), as well as on the virtual bone models using both software packages (in-house software K = 0.78–0.84 and 0.62–0.72; MeshLab K = 0.84–0.90 and 0.62–0.80). See Table 1 for the scores per trait and modality.

**Table 1:** Cohen’s weighted kappa (K) values for the levels of intra- and inter-observer agreement for Klales (2012) traits when scored on dry bones and virtual bone models

	VA	SPC	MA
	Kappa values for intra-observer agreement		
Dry bone	0.89	0.81	0.78
Virtual bone in-house software	0.84	0.84	0.78
Virtual bone MeshLab	0.90	0.86	0.84
	Kappa values for inter-observer agreement		
Dry bone	0.67	0.77	0.61
Virtual bone in-house software	0.72	0.69	0.62
Virtual bone MeshLab	0.80	0.68	0.62

*VA* ventral arc, *SPC* sub-pubic concavity, *MA* medial aspect ischial-pubic ramus

#### Agreement between the virtual bone models and their dry bone counterparts

The agreement between the Klales (2012) traits scored on the virtual bone models and their dry bone counterparts indicate substantial to almost perfect agreement for all features (K = 0.74–0.82) when using the in-house research software. The level of agreement was slightly less with moderate to substantial agreement (K = 0.58–0.76) when using MeshLab (see Table 2).

**Table 2:** Cohen’s weighted kappa (K) values indicating levels of agreement for Kales (2012) traits between virtual bone models and their dry bone counterparts

	VA	SPC	MA
	Kappa values virtual bone model vs. dry bone		
Virtual in-house software vs. dry bone	0.74	0.84	0.82
Virtual MeshLab vs. dry bone	0.58	0.76	0.70

*VA* ventral arc, *SPC* sub-pubic concavity, *MA* medial aspect ischial-pubic ramus

### Sex estimation features based on the WEA (1980) traits

#### Intra- and inter-observer agreement

The intra-observer agreement for the dry bone elements for all five WEA (1980) traits was substantial to almost perfect (K = 0.76–0.88). Similar values were found on the virtual bone models, using the in-house research software (K = 0.64–0.91 for all five traits). Using MeshLab, the scores of all but one trait (the IB) indicated almost perfect agreement (K = 0.82–0.86). The intra-observer agreement for the ischial body was “fair” (K = 0.40).

Results were less unambiguous for the inter-observer agreement. In the dry bone elements, Cohen’s weighted kappa values indicated substantial to almost perfect agreement for PAS, PA, and AC (K = 0.70–0.88). Inter-observer agreement for the GSN was moderate (K = 0.51) and for the IB fair (K = 0.24). In the virtual models (using both software packages), substantial to almost perfect inter-observer agreement was found for the PAS, GSN, and PA (in-house software K = 0.65–0.83, MeshLab K = 0.68–0.83), while the scores for AC indicated moderate agreement (K = 0.54 and K = 0.55). The IB performed worst with kappa values of 0.33 and 0.05 for the in-house software and MeshLab, respectively (see Table 3).

**Table 3:** Cohen’s weighted kappa (K) values indicating levels of intra- and inter-observer agreement for WEA (1980) traits when scored on dry bones and virtual bone models

	PAS	GSN	PA	AC	IB
	Kappa values for intra-observer agreement				
Dry bone	0.93	0.76	0.80	0.88	0.80
Virtual in-house software	0.71	0.91	0.80	0.81	0.64
Virtual MeshLab	0.86	0.82	0.84	0.83	0.40
	Kappa values for inter-observer agreement				
Dry bone	0.88	0.51	0.76	0.70	0.24
Virtual in-house software	0.65	0.83	0.82	0.55	0.33
Virtual MeshLab	0.80	0.68	0.83	0.54	0.05

*PAS* pre-auricular sulcus, *GSN* greater sciatic notch, *PA* pubic angle, *AC* arc compose, *IB* ischial body

#### Agreement between the virtual models and their dry bone counterparts

The agreement between the virtual bone models and their dry bone counterparts for the five WEA (1980) traits indicate substantial to almost perfect agreement (K = 0.68–0.85) for three of the five traits, namely the GSN, PA and AC when using the in-house research software (see Table 4). PAS and IB showed moderate agreement (K = 0.42–0.50). Using MeshLab, the scores of all but one trait (IB) indicated substantial to almost perfect agreement (K = 0.74–0.87). IB showed fair agreement (K = 0.33).

**Table 4:** Cohen’s weighted kappa (K) values indicating levels of agreement for WEA (1980) traits when scored on virtual bone models and their dry bone counterparts

	PAS	GSN	PA	AC	IB
	Kappa values virtual bone model vs. dry bone				
Virtual in-house software vs. dry bone	0.42	0.80	0.85	0.68	0.50
Virtual MeshLab vs. dry bone	0.81	0.74	0.87	0.80	0.33

*PAS* pre-auricular sulcus, *GSN* greater sciatic notch, *PA* pubic angle, *AC* arc compose, *IB* ischial body

**Fig. 2:**
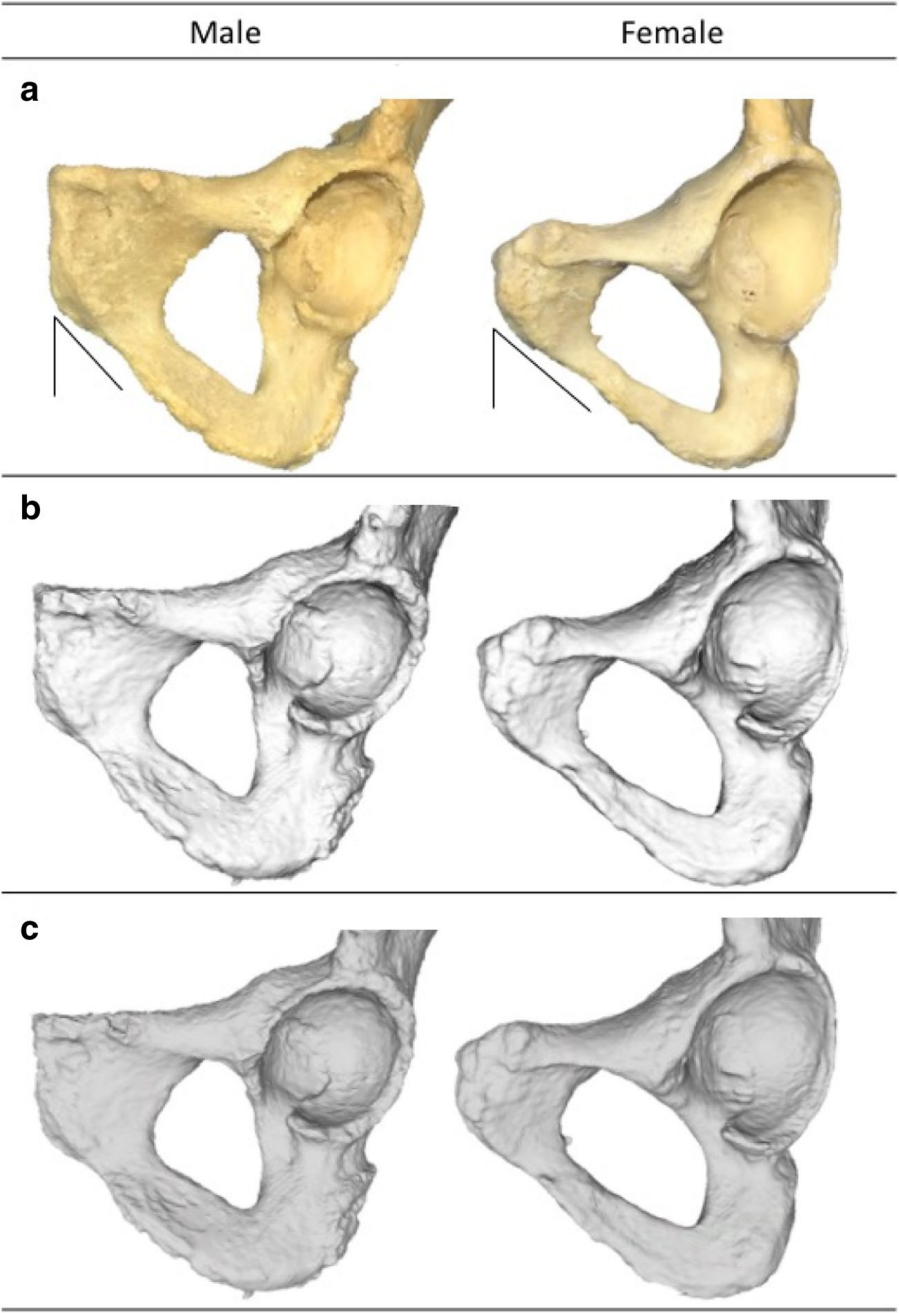
A male and female example of a trait that performed reliably is the pubic angle on dry bone (**a**) and on two virtual 3D models; the in-house research software package (**b**) and MeshLab (**c**). The pubic angle is indicated by the lines in **a**

**Fig. 3:**
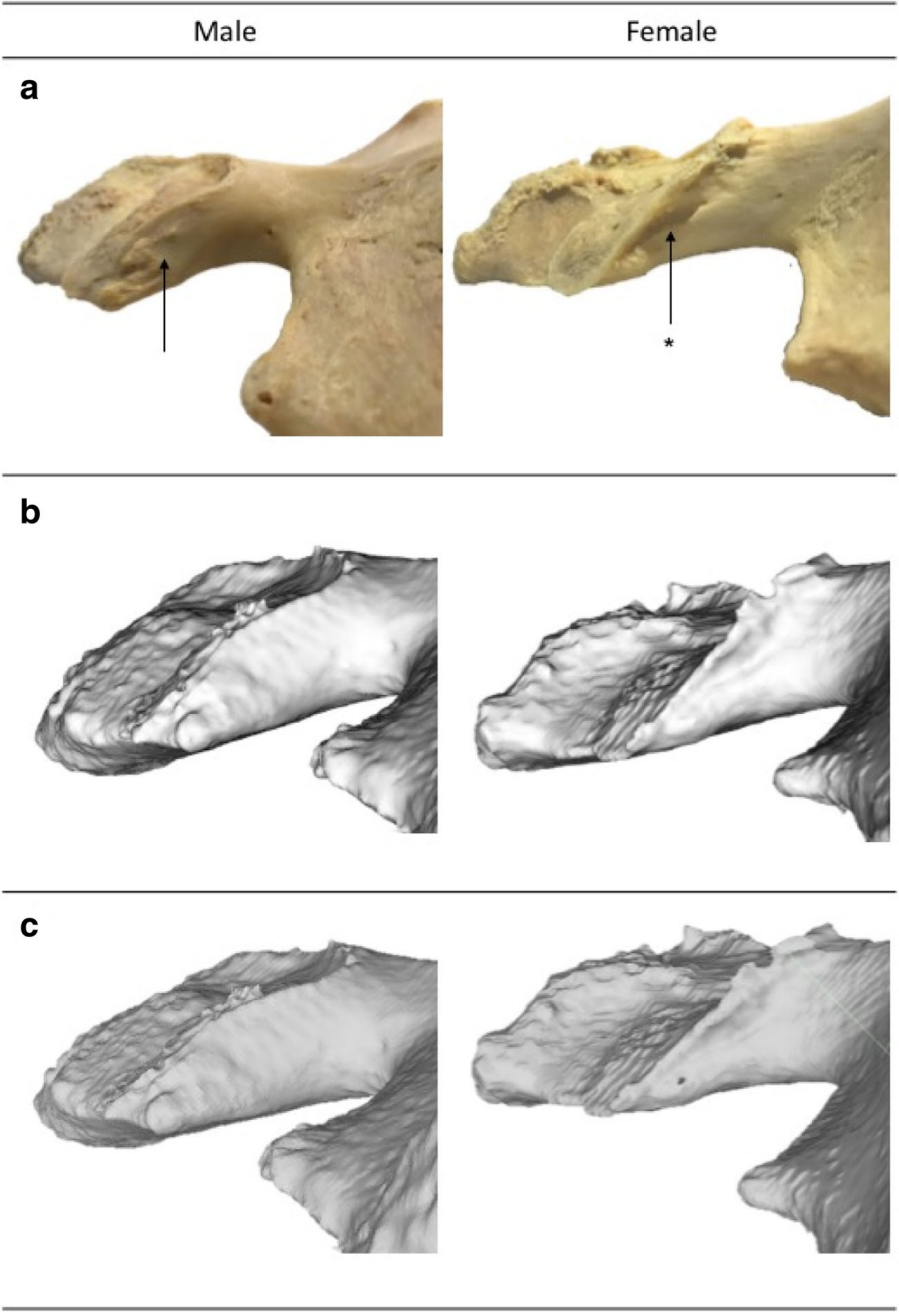
A male and female example of a trait that performed poorly is the pre-auricular sulcus, on dry bone (**a**) and on virtual 3D models; the in-house research software package (**b**) and MeshLab (**c**). The arrows indicate the region of interest with the sulcus being present (*) on the female pelvis. Note that the pre-auricular sulcus is clearly visible on the dry bone, but invisible when viewed with the in-house software and poorly defined when looking at the reconstruction with MeshLab

An example of the differences/similarities observed when comparing the dry bone- and virtual 3D models is demonstrated in Fig. 2, exemplifying a trait that scored well (pubic angle), and Fig. 3, illustrating the differences observed in a feature that performed poorly (pre-auricular sulcus).

## Discussion

Intra- and inter-observer agreement results indicate that all three Klales (2012) traits can be scored reliably on both the virtual bone models (regardless of which viewer software package is used) and on the dry bone counterparts. Notable is that the intra- and inter-observer agreement found in this study were comparable to those in the original Klales (2012) publication [8]. Furthermore, the results from this study are in line with previous research conducted by Johnstone-Belford (2018) [24], which found that the intra-observer variation is limited when the binary Phenice traits [35] are scored on virtual bone models. Additionally, scores assigned for all three Klales (2012) traits on the virtual bone models showed acceptable accuracy scores. VA shows slightly reduced accuracy when viewed in MeshLab, but the kappa value of 0.58 is still considered acceptable by the authors. Based on these results, the authors conclude that the Klales (2012) morphological sex estimation technique can be used interchangeably between virtual bone models and dry skeletal elements. Consequently, classification accuracies of the published method should be valid on virtual bone models and additionally, virtual data from clinical CT scans can be used to determine population specific accuracies.

The same does not hold for all selected traits incorporated in the WEA (1980) morphological sex estimation technique.

The IB scored poorly overall and proved to be a difficult trait to score on both the virtual bone models and the dry bones. In this study, the IB was found to be neither reliable (i.e. lack of intra- and inter-observer agreement) or accurate (i.e. lack of agreement between the scores in the virtual bone model-dry bone pair); thus, the authors feel that this should prompt a reconsideration of the IB as a sex estimation trait.

Intra-observer agreement for the four remaining WEA traits was substantial for both the virtual bone models (regardless of which viewer software package is used) and dry bone counterparts. However, inter-observer agreement scores showed that only the PAS and the PA show acceptable inter-observer agreement on the dry bone counterparts and on both the virtual bone models (regardless of which viewer software package is used). Inter-observer agreement levels were not acceptable for the GSN on dry bone and for the AC on virtual bone models (using both viewer software packages). Due to the lack of literature in which the intra- and inter-observer errors for each WEA (1980) trait is reported individually, the authors were unable to compare the presented results to previous reliability performances of the separate traits.

Based on the agreement of scores between the virtual bone model-dry bone pairs, the sex estimation scores on virtual bone models for the GSN, PA and AC accurately correspond to those on the dry skeletal element (regardless of which viewer software package is used). However, only the PA can be used interchangeably between virtual models and dry skeletal elements, since the GSN and AC are disqualified on the basis of their lack of inter-observer agreement.

Although the PAS performed reliably (i.e. intra- and inter-observer agreement), conflicting accuracy (i.e. virtual bone model-dry bone pair agreement between scores) results were found when using different viewing software packages. Apparently, the use of a specific viewer software package has a substantial effect on the visualization of this trait (Fig. 2). A potential explanation might be that the scoring of the PAS on dry bones traditionally involves palpating the region of interest and using a slanted light source to view the depth of the sulcus. The in-house research software does not provide an artificial light source to visualize the sulcus, thereby hampering the sex estimation. In contrast, the agreement scores increased when using MeshLab, which does provide a light source.

For the WEA (1980) traits, it can be concluded that only the PA can be scored reliably and accurately on virtual bone models, regardless of the viewing software. If MeshLab is used as a viewing software, then PAS also performs reliably and accurately. This means that the classification accuracies of these traits can be applied to virtual bone models and that clinical CT scans can be used as a source for the development of their population-specific accuracies.

In this study, a single segmentation method was used, which may be considered a limitation since a different segmentation method may result in slightly different virtual bone models [36]. Since these differences are generally small, it is unlikely that this would have an effect on scoring morphological traits. It is important to note that this study focusses on the reliability and accuracy of the sex estimation traits and not on the reliability and accuracy of sex estimation itself. The latter would require a much larger study population with a less skewed age distribution. The skewed age distribution might initially be viewed as a limitation of the study, however, this is nullified by the aim of the current study. As a matter of fact, the relatively old age of the used population presents somewhat of a “worst case scenario” since the aging effect on joint surfaces and areas of muscle attachments might interfere with the segmentation process and consequently the anthropological analysis. It is therefore expected that the studied traits will perform similarly or better in younger individuals. Another limitation may be the use of only five WEA traits, however as previously mentioned, these five were chosen specifically for the range of complexity that they provide.

This study assumes that the maceration process did not influence the morphology of the scored Klales (2012) and WEA (1980) traits. This is based on the absence of any published data on a potential change in these traits due to the maceration process. Also, since the dry bone elements were taken as the golden standard to which the virtual bone models were compared, this potential change in shape does not influence the results of this study.

The findings in this study further support the recently initiated and progressing shift towards virtual forensic anthropology, but also reveals limitations regarding the applicability of certain traits to virtual bone models. To our knowledge, this is the first study that investigates the accuracy of individual morphological traits on virtual bone models in comparison to their dry bone counterparts. Previous studies have investigated the accuracy of virtual bones; however, these studies did so by using metric sex estimation methods [30, 37, 38].

The authors believe this study to be a useful addition to current literature, as it illustrates the feasibility of using virtual bone models, derived from clinical CT scans, as a proxy for reference populations, while simultaneously showing that caution is advised when interchangeably applying traditional forensic anthropological methodology on virtual bone models. As demonstrated in this study, not all traits within a given technique achieve the same level of agreement. Thus, it is essential to study the agreement between the traits of interest before applying morphological sex estimation techniques interchangeably on virtual bone models and the dry skeletal elements.

## Conclusion

The Klales (2012) morphological sex estimation technique is as reliable on virtual bone models (regardless of which viewer software package is used) as it is on dry pelvic bones. Furthermore, the sex estimation scores on the virtual bone models agree sufficiently with those on dry bone. The same does not hold for the five investigated WEA (1980) morphological sex estimation traits which show almost invariably limited reliability and/or limited accuracy. Only the PA (and the PAS when viewed in MeshLab) showed acceptable results in terms of reliability and accuracy.

This study shows that clinical CT data can be used as a source for contemporary and population-specific forensic anthropological data, but that the agreement between virtual bone models and the dry skeletal elements should always be considered before forensic anthropological morphological methods are applied interchangeably.
